# Standardized* Kaempferia parviflora* Extract Inhibits Intrinsic Aging Process in Human Dermal Fibroblasts and Hairless Mice by Inhibiting Cellular Senescence and Mitochondrial Dysfunction

**DOI:** 10.1155/2017/6861085

**Published:** 2017-08-02

**Authors:** Ji-Eun Park, Seon Wook Woo, Mi-Bo Kim, Changhee Kim, Jae-Kwan Hwang

**Affiliations:** Department of Biotechnology, College of Life Science and Biotechnology, Yonsei University, Seoul 03722, Republic of Korea

## Abstract

Intrinsic skin aging is a complex biological phenomenon mainly caused by cellular senescence and mitochondrial dysfunction. This study evaluated the inhibitory effect of* Kaempferia parviflora* Wall ex. Baker ethanol extract (KPE) on H_2_O_2_-stimulated cellular senescence and mitochondrial dysfunction both in vitro and in vivo. KPE significantly increased cell growth and suppressed senescence-associated *β*-galactosidase activation. KPE inhibited the expression of cell-cycle inhibitors (p53, p21, p16, and pRb) and stimulated the expression of cell-cycle activators (E2F1 and E2F2). H_2_O_2_-induced hyperactivation of the phosphatidylinositol 3-kinase/protein kinase B (AKT) signaling pathway was suppressed by KPE through regulated expression of forkhead box O3a (FoxO3a) and mammalian target of rapamycin (mTOR). KPE attenuated inflammatory mediators (interleukin-6 (IL-6), IL-8, nuclear factor kappa B (NF-*κ*B), and cyclooxygenase-2 (COX-2)) and increased the mRNA expression of PGC-1*α*, ERR*α*, NRF1, and Tfam, which modulate mitochondrial biogenesis and function. Consequently, reduced ATP levels and increased ROS level were also reversed by KPE treatment. In hairless mice, KPE inhibited wrinkle formation, skin atrophy, and loss of elasticity by increasing the collagen and elastic fibers. The results indicate that KPE prevents intrinsic aging process in hairless mice by inhibiting cellular senescence and mitochondrial dysfunction, suggesting its potential as a natural antiaging agent.

## 1. Introduction

Intrinsic skin aging is a complex biological phenomenon mainly caused by intracellular stressors [1]. Among various factors that accelerate intrinsic aging, the major causes are cellular senescence and mitochondrial dysfunction [2]. When proliferating cells are exposed to various types of stressors, they may undergo growth arrest, termed as cellular senescence [3]. Senescent cells exhibit various phenomena, such as cell-cycle arrest, gene expression changes, and secretion of inflammatory cytokines [4]. Reactive oxygen species (ROS) mainly accelerate cellular senescence and also play a role in determining the lifespan of mammalian cells [1]. Although senescent cells are relatively rare in young organisms, their number increases according to the aging. The most obvious sign of cellular senescence is cell-cycle arrest in the G1 phase with an altered gene expression pattern [3]. Senescent cells secrete numerous factors that have harmful effects on cells. As p16 and p21 are cyclin-dependent kinase inhibitors (CDKIs), two major pathways, the p53/p21 pathway and p16/retinoblastoma protein (pRb) pathway, regulate cell-cycle arrest. Two transcriptional regulators, p53 and pRb, mediate the activities of p21 and p16, respectively [5].

The phosphatidyl inositol-3 kinase (PI3K)/AKT signaling pathway is known to modulate cellular senescence and longevity in many organisms [6]. Oxidative stress-induced cellular senescence stimulates the PI3K/AKT pathway, subsequently suppressing forkhead box O (FoxO) transcriptional factors and elevated mammalian target of rapamycin (mTOR) [7]. In addition, the accumulation of senescent cells with aging affects neighboring cells resulting in tissue damage and inflammation. The presence of many inflammatory cytokines and mediators secreted by senescent cells can destroy tissue structures and functions [8].

Mitochondria also play a critical role in aging. During the aging process, mitochondria lose their function, leading to a decline in mitochondrial biogenesis and ATP production [9]. Mitochondrial dysfunction or impaired mitochondrial activity can contribute to imbalance in energetic metabolism and oxidative stress resulting in ROS accumulation. ROS are generated as by-products of ATP synthesis in mitochondria [10]. Excessively accumulated ROS negatively influence various cellular components such as proteins, lipids, and nucleic acids, leading to age-associated phenotypes and diseases [11]. Accordingly, antiaging is associated with mitochondrial homeostasis [12]. Peroxisome proliferator-activated receptor *γ* coactivator 1 *α* (PGC-1*α*) is a key activator of mitochondrial biogenesis and function by stimulating stimulates downstream factors such as estrogen-related receptor alpha (ERR*α*), nuclear respiratory factor 1 (NRF1), and mitochondrial transcription factor A (Tfam) responsible for replication, transcription, and translation of mitochondrial DNA (mtDNA) [12]. Thus, a low level of PGC-1*α* gives rise to mitochondrial dysfunction and aging.

*Kaempferia parviflora* Wall. ex Baker, commonly called black ginger, has been used as a dietary supplement and traditional medicine in tropical countries [13].* K. parviflora* is reported to have antioxidative, anti-inflammatory, antiviral, and anticancer activities [13–15]. However, its effect on intrinsic skin aging has not been verified. We investigated the inhibitory effect of* K. parviflora* on intrinsic skin aging process by evaluating its effect on cellular senescence and mitochondrial dysfunction using H_2_O_2_-exposed human dermal fibroblasts. In addition, its effect on skin aging phenotypes was evaluated using hairless mice.

## 2. Materials and Methods

### 2.1. Preparation of Standardized* K. parviflora* Extract (KPE)

Rhizomes of* K. parviflora *were collected in Bangkok, Thailand. The dried rhizomes were ground and extracted with 95% ethanol in a shaking water bath at 60°C for 3 h. Then, the extract was concentrated using a rotary evaporator (Heidolph Instruments GmbH & Co. KG., Schwabach, Germany) to obtain the KPE with a yield of 8.9% (w/w), including 14.1% (w/w) 5,7-dimethoxyflavone as a bioactive compound [16].

### 2.2. Cell Culture and H_2_O_2_ Exposure

Human dermal fibroblasts (Hs68) purchased from the American Type Culture Collection (Manassas, VA, USA) were cultured in Dulbecco's modified Eagle's medium (DMEM; Hyclone laboratories, Logan, UT, USA) supplemented with penicillin (120 units/ml), streptomycin (75 *μ*g/ml), and 10% fetal bovine serum (FBS; Gibco, Rockville, MD, USA) at 37°C with 5% CO_2_. At 70% confluence, the cells were treated with 600 *μ*M H_2_O_2_ (Sigma-Aldrich, St. Louis, MO, USA) for 2 h to induce a senescent state.

### 2.3. Animal Experiments

Eight-week-old female hairless mice (SKH-1: Orient Bio Inc., Seongnam, Korea) were housed in temperature- (23 ± 2°C), humidity- (55 ± 10%), and light- (12 h day/12 h night) controlled conditions for 24 weeks at Yonsei Laboratory Animal Research Center (YLARC; Seoul, Korea). All experimental protocols were approved by the Institutional Animal Care and Use Committee (IACUC) of YLARC (Permit number is 2013-0113). Mice were randomly assigned into two groups: (1) middle-aged (MA) group and (2) MA administered with KPE group. Mice in the KPE administered group orally received 200 mg/kg/day KPE for 24 weeks. For the young group, 7-week-old female hairless mice were purchased from Orient Bio Inc. (Seongnam, Korea) at 1 week before they were killed and were allowed to acclimatize for 1 week. After 24 weeks, the mice were killed under anesthesia by using an intraperitoneal injection of a mixture of zoletil (Virbac, Carros, France) and rompun (Bayer Korea Ltd., Seoul, Korea). Sample from the dorsal skin were rapidly frozen in liquid nitrogen and stored at −70°C. Skin biopsy samples were fixed in 10% buffered formalin for optical microscopy in order to analyze histological changes. All efforts were made to minimize the suffering of mice.

### 2.4. Cell Growth Assay

Cell growth was evaluated using an MTT assay [3-(4,5-dimethylthiazol-2-yl)-2,5-diphenyltetrazolium bromide; Sigma-Aldrich]. The cells cultured in 96-well plates for 24 h were pretreated with the samples (KPE or resveratrol) for 24 h in a serum-free medium and were subsequently exposed to 600 *μ*M H_2_O_2_ in order to induce growth arrest. After a further 72 h of treatment, the culture medium was replaced with MTT solution (0.1 mg/ml) and incubated in darkness for an additional 3 h. The insoluble formazan products were dissolved in dimethyl sulfoxide (DMSO) and were measured using a VersaMax tunable microplate reader (Molecular Devices, Sunnyvale, CA, USA) at 540 nm for absorbance.

### 2.5. Senescence-Associated *β*-Galactosidase Assay

The cellular SA-*β*-gal activity was evaluated using a 96-well cellular senescence assay kit (Cell Biolabs, San Diego, CA, USA), following the described assay protocol. The whole lysate of cells treated with cold 1x cell lysis buffer was centrifuged for 10 min at 4°C. Next, 50 *μ*l of the supernatant was mixed with an equal amount of 2x assay buffer and incubated at 37°C for 1 h. The activity of SA-*β*-gal was then measured using a fluorescence plate reader (GloMax® Multi Microplate Reader; Promega, Madison, WI, USA) at 360 nm (excitation)/465 nm (emission).

### 2.6. Reverse Transcription-Polymerase Chain Reaction (RT-PCR)

The dorsal skin of hairless mice was grinded using mortar and pestle in liquid nitrogen and then it was homogenized in Trizol reagent for RT-PCR analysis. PCR amplification of cDNA products (3 *µ*l) was performed using a PCR premix and the following primer pairs (Bioneer, Daejeon, Korea): human p53 (forward, 5′-ACA CGC TTC CCT GGA TTG G-3′; reverse, 5′-CTG GCA TTC TGG GAG CTT CA-3′), human p21 (forward, 5′-GTC AGT TCC TTG TGG AGC CG-3′; reverse, 5′-GGA AGG TAG AGC TTG GGC AG-3′), human p16 (forward, 5′-GGG TCC CAG TCT GCA GTT AAG-3′; reverse, 5′-CAG TTG GTC CTT CTC GGT CC-3′), human pRb (forward, 5′-TTT ATT GGC GTG CGC TCT TG-3′; reverse, 5′-CAG TAG CAT CAG CAC GAG GG-3′), human E2F1 (forward, 5′-CCG CCA TCC AGG AAA AGG TG-3′; reverse, 5′-GCT ACG AAG GTC CTG ACA CG-3′), human E2F2 (forward, 5′-GAC TAG AGA GCG AGC CGC AA-3′; reverse, 5′-GAG CAG AGA GCA GCG CTT AG-3′), human SIRT1 (forward, 5′-ACC GAG ATA ACC TTC TGT TCG-3′, reverse, 5′-CAC CCC AGC TCC AGT TAG AA-3′), human IL-6 (forward, 5′-ATG AGG AGA CTT GCC TGG TG-3′; reverse, 5′-ACA ACA ATC TGA GGT GCC CA-3′), human IL-8 (forward, 5′-CCA GGA AGA AAC CAC CGG AA-3′; reverse, 5′-CCT CTG CAC CCA GTT TTC CT-3′), human PGC-1*α* (forward, 5′-GTG AAG GGC AAG CCA CTC TG-3′; reverse, 5′-GGT CTT CAC CAA CCA GAG CA-3′), human NRF-1 (forward, 5′-GGT GTG ATA AAC CCC ATT TCA CC-3′; reverse, 5′-AGT GGC AAG GCA GTG AAT GA-3′), human Tfam (forward, 5′-AGC TCA GAA CCC AGA TGC AAA-3′, reverse, 5′-TTC AGC TTT TCC TGC GGT GA-3′), human ERR*α* (forward, 5′-ATG GTG TGG CAT CCT GTG AG-3′; reverse, 5′-ATT CAC TGG GGC TGC TGT C-3′), human GAPDH (forward, 5′-CTC CTG TTC GAC AGT CAG CC-3′; reverse, 5′-TCG CCC CAC TTG ATT TTG GA-3′) mouse p53 (forward, 5′-CTT GGC TGT AGG TAG CGA CT-3′; reverse, 5′-CAG CAA CAG ATC GTC CAT GC-3′), mouse p21 (forward, 5′-CGG TGT CAG AGT CTA GGG GA-3′; reverse, 5′-AGG CCA TCC TCA AAT GGT GG-3′), mouse p16 (forward, 5′-CGC AGG TTC TTG GTC ACT GT-3′; reverse, 5′-CTG CAC CGT AGT TGA GCA GA-3′), mouse pRb (forward, 5′-TTT TGT AAC GGG AGT CGG GT-3′; reverse, 5′-AAG ATG CAG ATG CCC CAG AG-3′), mouse PGC-1*α* (forward, 5′-GTC CTT CCT CCA TGC CTG AC-3′; reverse, 5′- GAC TGC GGT TGT GTA TGG GA-3′), mouse NRF-1 (forward, 5′- CTT CAT GGA GGA GCA CGG AG-3′; reverse, 5′-ATG AGG CCG TTT CCG TTT CT-3′), mouse Tfam (forward, 5′- ATA GGC ACC GTA TTG CGT GA-3′, reverse, 5′-CTG ATA GAC GAG GGG ATG CG-3′), mouse ERR*α* (forward, 5′-GCC CAT GCA CAA GCT GTT TT-3′; reverse, 5′- ACA CAC AAA GTG GGG AGG GA-3′), mouse *β*-actin (forward, 5′-AGG ATC TTC ATG AGG TAG T-3′; reverse, 5′-GCT CCG GCA TGT GCA A-3′). Before PCR amplification, the primers were denatured at 94°C for 5 min. Amplification comprised 24–30 cycles of denaturation at 94°C for 30 s, annealing at 54–59°C for 1 min, extension at 72°C for 1 min, and then a final extension for 5 min at 72°C. PCR was performed in the Gene Amp PCR System 2700 (Applied Biosystems, Foster City, CA, USA). The PCR products were then separated via 1.5% agarose gel electrophoresis and visualized via 6x Loading STAR solution and UV illumination using the G:BOX Image Analysis System (Syngene, Cambridge, UK).

### 2.7. Western Blot Analysis

Cultured Hs68 cells were lysed in NP-40 buffer (ELPIS-Biotech, Daejeon, Korea) with a protease inhibitor cocktail (Sigma-Aldrich). The dorsal skin of hairless mice was grinded using mortar and pestle in liquid nitrogen and then it was homogenized in NP-40 buffer (ELPIS-Biotech) with a protease inhibitor cocktail (Sigma-Aldrich). Protein concentrations were determined using the Bradford assay. For western blot analysis, 20 *μ*g of protein samples was separated via 10% sodium dodecyl sulfate-polyacrylamide gel electrophoresis (SDS-PAGE), transferred to membranes, and blocked with 5% skim milk in Tris-buffered saline with Tween-20 (TBST). The membranes were then probed with primary antibodies against p53, p21, p16, pRb, PI3K, phospho-AKT, AKT, phospho-FoxO3a, FoxO3a, phospho-mTOR, mTOR, SIRT1, NF-*κ*B, COX-2, PGC-1*α*, and *α*-tubulin (Cell Signaling, Beverly, MA, USA). Bound antibodies were detected with a horseradish peroxidase-linked secondary antibody (Bethyl Laboratories, Montgomery, TX, USA). Bound antibody signals were detected using an ECL detection solution (Amersham ECL Western Blotting Detection Reagents; GE Healthcare, Uppsala, Sweden) and were visualized using the G:BOX Image Analysis System (Syngene).

### 2.8. Evaluation of Skin Wrinkle Formation

The dorsal skin surface of anesthetized hairless mice was analyzed with replica (Epigem, Seoul, Korea) and Visioline VL650 (CK Electronics GmbH, Cologne, Germany).

### 2.9. Histological Analysis

The skin samples fixed in 10% formalin for 24 h were sectioned and stained with hematoxylin and eosin (H&E), Masson's trichrome (M&T), and Verhoeff-van Gieson's stain (EVG). The stained skin samples were analyzed using an Eclipse TE2000U Inverted Microscope with twin CCD cameras (Nikon, Tokyo, Japan).

### 2.10. Hydroxyproline Assay

Skin tissues (100 mg) were homogenized using glass beads in 1 mL of 6 N HCl and were hydrolyzed at 105°C for 20 h [13]. The hydroxyproline content was then measured using a hydroxyproline assay kit (QuickZyme Biosciences, Leiden, Netherlands).

### 2.11. Evaluation of Skin Elasticity

Skin elasticity was evaluated using Cutometer® MPA580 (CK Electronics GmbH). Among the parameters, *R*2 was used as the main parameter to assess skin elasticity and skin aging.

### 2.12. Statistical Analysis

Results are expressed as the mean ± standard deviation (SD) of triplicate experiments. All groups were compared via one-way analysis of variance (ANOVA), followed by Scheffe's test using SPSS 21 software (Chicago, IL, USA). *p* values less than 0.05 were marked and considered statistically significant: ^#^*p *< 0.05 and ^##^*p *< 0.01 (normal versus H_2_O_2_ control and young versus MA group); ^*∗*^*p* < 0.05 and ^*∗∗*^*p* < 0.01 (H_2_O_2_ control versus sample-treated cells and young versus MA group).

## 3. Results

### 3.1. Effect of KPE on Cell Growth In Vitro

**Figure 1:**
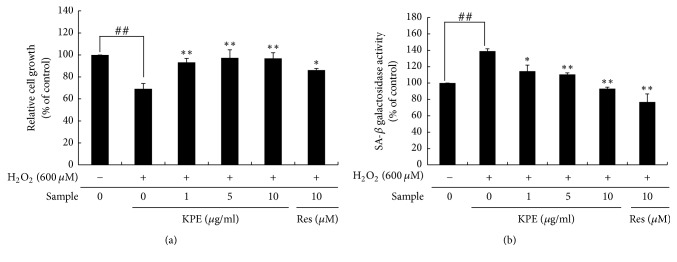
Effect of KPE on cell growth promotion and SA-*β*-gal activity. Hs68 cells were pretreated with KPE (1–10 *μ*g/ml) or resveratrol (10 *μ*M) for 24 h. After 2 h of H_2_O_2_ exposure, the cells were cultured for an additional 72 h. (a) The number of proliferating cells was measured using an MTT assay. (b) The cellular SA-*β*-gal activity was detected using a fluorescence reader at 365 nm excitation and 460 nm emission. Data are represented as mean ± SD from triplicate independent experiments. ^##^*p *< 0.01 (normal versus H_2_O_2_ control); ^*∗*^*p* < 0.05 and ^*∗∗*^*p* < 0.01 (H_2_O_2_ control versus sample-treated cells).

Cellular senescence inhibits cell proliferation and decreases the number of cells [17]. H_2_O_2_ exposure reduced cell proliferation compared to the normal cells; however, KPE treatment significantly reinstated the proliferative activity of Hs68 cells to almost the normal level (Figure 1(a)). KPE at 1, 5, and 10 *μ*g/ml showed 1.35-, 1.41-, and 1.40-fold increase in relative cell growth, respectively, compared to that in the H_2_O_2_ control.

### 3.2. Effect of KPE on H_2_O_2_-Induced SA-*β*-Gal Expression In Vitro

Senescent cells are featured by the activation of SA-*β*-gal, the most important biomarker to identify cellular senescence [3, 18]. H_2_O_2_ exposure caused almost a 1.39-fold increase in the SA-*β*-gal activity in Hs68 cells, but this activity was dose-dependently decreased by KPE (Figure 1(b)). The relative SA-*β*-gal activity after treatment with 1, 5, and 10 *μ*g/ml KPE showed a 17.6%, 20.5%, and 33.0% reduction, respectively, compared to that in the H_2_O_2_ control.

### 3.3. Effect of KPE on H_2_O_2_-Induced Cell-Cycle Arrest In Vitro

**Figure 2:**
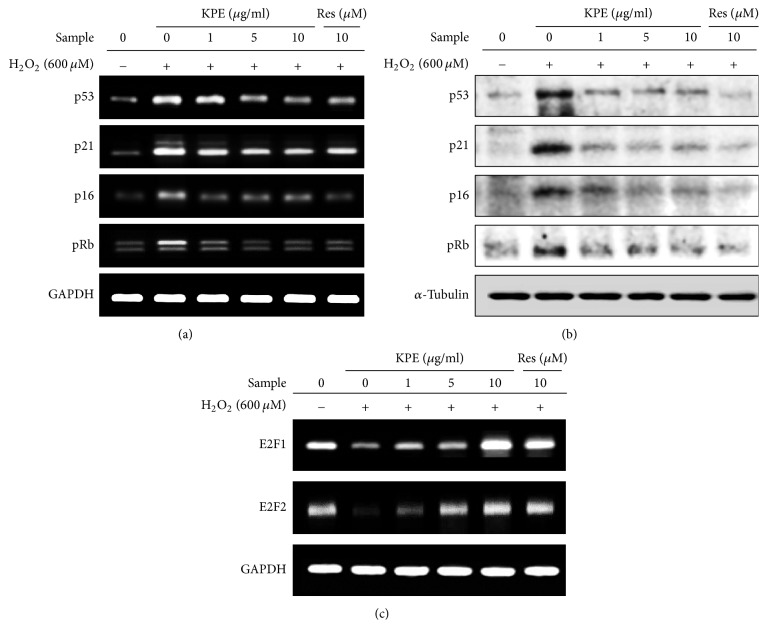
Effect of KPE on cell-cycle arrest. Hs68 cells were pretreated with KPE (1–10 *μ*g/ml) or resveratrol (10 *μ*M) for 24 h. After 2 h of H_2_O_2_ exposure, the cells were cultured for an additional 24 h. (a, b) mRNA and protein levels of cell-cycle inhibitors (p16, p21, p53, and pRb) were investigated via RT-PCR and western blotting, respectively. (c) mRNA expression of cell-cycle activators, E2F1 and E2F2, was investigated via RT-PCR. Equal loading of mRNA and protein was verified based on GAPDH and *α*-tubulin levels, respectively.

Cell-cycle arrest in the senescent state results from alteration of markers associated with the cell cycle, including cell-cycle inhibitors (p53, p21, p16, and pRb) and cell-cycle activators (E2F1 and E2F2) [5]. The mRNA levels of cell-cycle inhibitors were significantly reduced by KPE treatment (Figure 2(a)). The protein expression of cell-cycle inhibitors was dose-dependently reduced upon KPE treatment (Figure 2(b)). The mRNA expression of cell-cycle activators was downregulated upon H_2_O_2_ exposure but was highly elevated by KPE (Figure 2(c)).

### 3.4. Effect of KPE on H_2_O_2_-Induced Activation of the PI3K/AKT Pathway In Vitro

**Figure 3:**
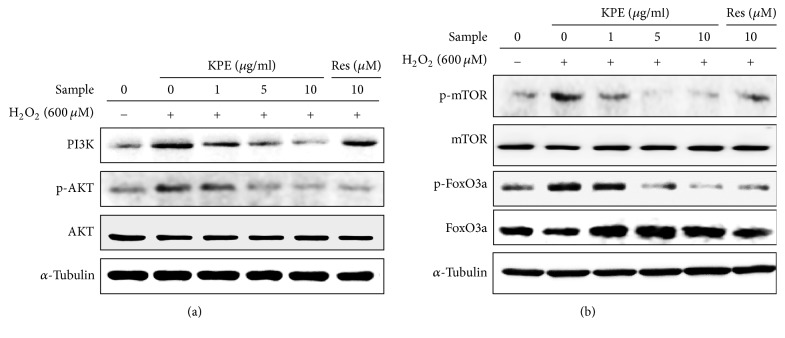
Effect of KPE on the PI3K/AKT pathway. Hs68 cells were pretreated with KPE (1–10 *μ*g/ml) or resveratrol (10 *μ*M) for 24 h. After 2 h of H_2_O_2_ exposure, the cells were cultured for an additional 24 h. Protein expression of associated markers in the PI3K/AKT signaling pathway was evaluated via western blotting. The effect of KPE on (a) PI3K and AKT activation and (b) the levels of mTOR and FoxO3a are shown. *α*-Tubulin was used as the loading control.

Oxidative stress activates the PI3K/AKT pathway, triggering cellular senescence. PI3K/AKT downstream markers such as FoxO3a and mTOR are also intimately associated with cellular senescence [7]. The H_2_O_2_-induced expression of PI3K and phospho-AKT was decreased by KPE without any visible changes in the total AKT level compared to that in the H_2_O_2_ control (Figure 3(a)). In addition, KPE treatment increased FoxO3a (an active form) and decreased phospho-FoxO3a (an inactive form) levels. Phospho-mTOR protein expression was increased by H_2_O_2_-induced cellular senescence, but KPE significantly reduced its expression without changing the level of total mTOR (Figure 3(b)).

### 3.5. Effect of KPE on SIRT1 Expression In Vitro

**Figure 4:**
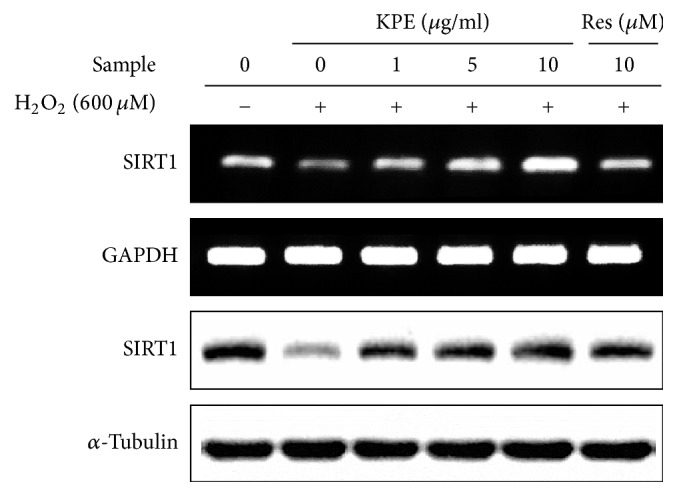
Effect of KPE on SIRT1 expression. Hs68 cells were pretreated with KPE (1–10 *μ*g/ml) or resveratrol (10 *μ*M) for 24 h. After 2 h of H_2_O_2_ exposure, the cells were cultured for an additional 24 h. SIRT1 mRNA and protein levels were determined via RT-PCR and western blotting, respectively. GAPDH and *α*-tubulin were used as the mRNA and protein loading controls, respectively.

SIRT1 is known as an effective antiaging factor that plays a central role in cellular senescence [4]. SIRT1 mRNA expression was significantly upregulated by KPE compared to that in the H_2_O_2_ control. The protein level of SIRT1 was also increased in KPE treated Hs68 cells (Figure 4).

### 3.6. Effect of KPE on H_2_O_2_-Induced Inflammatory Responses In Vitro

**Figure 5:**
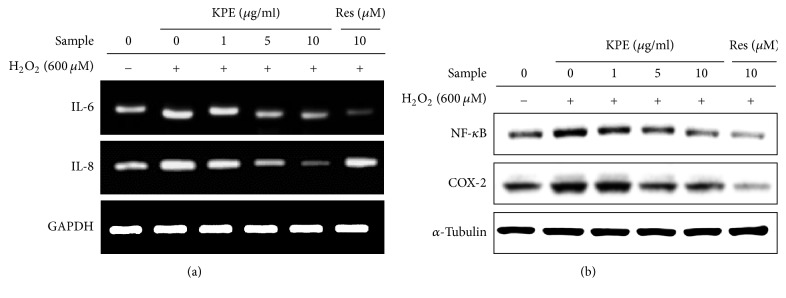
Effect of KPE on inflammatory mediators. Hs68 cells were pretreated with KPE (1–10 *μ*g/ml) or resveratrol (10 *μ*M) for 24 h. After 2 h of H_2_O_2_ exposure, the cells were cultured for additional 24 h. (a) Transcriptional changes in IL-6 and IL-8 were detected via RT-PCR. (b) Protein levels of NF-*κ*B and COX-2 were evaluated via western blotting. GAPDH and *α*-tubulin were used as the mRNA and protein loading controls, respectively.

Senescent cells show various increased senescence-associated secretory phenotypes (SASP), including cytokines, and other factors [8]. Senescence-associated inflammatory markers including interleukin-6 (IL-6), IL-8, nuclear factor kappa B (NF-*κ*B), and cyclooxygenase-2 (COX-2) were investigated to verify the anti-inflammatory effect of KPE in senescent fibroblasts. H_2_O_2_-induced senescent Hs68 cells exhibited higher IL-6 and IL-8 mRNA levels than that in normal cells; however, KPE treatment markedly reduced these mRNA levels (Figure 5(a)). Additionally, the protein expression of other inflammatory markers, NF-*κ*B and COX-2, was also decreased by KPE treatment as compared to that in the H_2_O_2_ control (Figure 5(b)).

### 3.7. KPE Reduces H_2_O_2_-Induced Mitochondrial Dysfunction In Vitro

**Figure 6:**
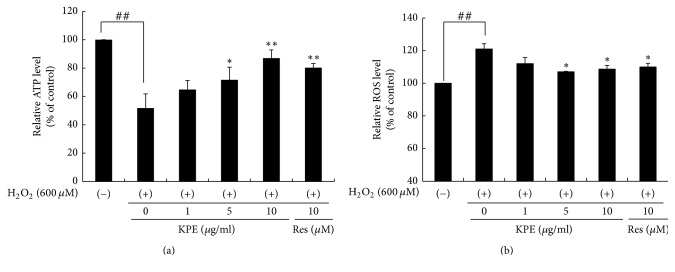
Effects of KPE on ATP and ROS production in vitro. Hs68 cells were pretreated with KPE (1–10 *μ*g/ml) or resveratrol (10 *μ*M) for 24 h. After 2 h of H_2_O_2_ exposure, the cells were cultured for an additional 24 h. (a) Effect of KPE on ATP production. (b) Effect of KPE on ROS production. Data are represented as mean ± SD of triplicate independent experiments. ^##^*p *< 0.01 compared to the nontreated control; ^*∗*^*p* < 0.05 and ^*∗∗*^*p* < 0.01 compared to the H_2_O_2_-treated control.

ATP and ROS levels were measured after H_2_O_2_ treatment to elucidate the effect of KPE on mitochondrial dysfunction. H_2_O_2_ markedly reduced the ATP levels compared with the untreated control; however, KPE restored the reduced ATP levels. The relative ATP level after treatment with 10 *μ*g/mL KPE increased by 35.3% as compared to that in the H_2_O_2_ control. On the contrary, maintenance of ROS levels is important for mitochondrial function. KPE treated cells showed 13% reduction in ROS levels compared to those in the H_2_O_2_ control (Figure 6).

### 3.8. KPE Increases Mitochondrial Biogenesis Transcription Factor Expression In Vitro

**Figure 7:**
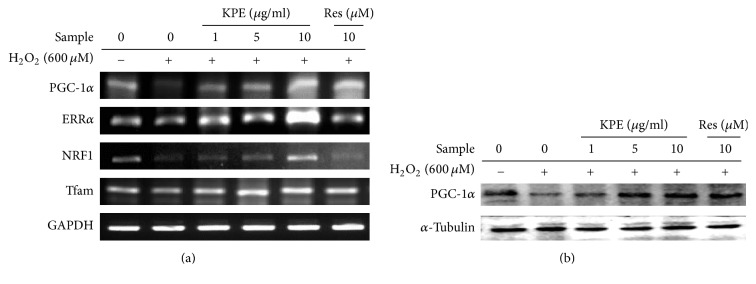
Effect of KPE on mitochondrial biogenesis transcription factor expression in vitro. Hs68 cells were pretreated with KPE (1–10 *μ*g/ml) or resveratrol (10 *μ*M) for 24 h in a serum-free medium. After 2 h of H_2_O_2_ exposure, the cells were cultured for additional 24 h. (a) Transcriptional changes in PGC-1*α*, ERR*α*, NRF-1, and Tfam were detected via RT-PCR. (b) Protein levels of PGC-1*α* were evaluated via western blotting. GAPDH and *α*-tubulin were used as the mRNA and protein loading controls, respectively.

To clarify whether KPE treatment regulates mitochondrial biogenesis transcription factors, the mRNA expression of PGC-1*α*, ERR*α*, NRF1, and Tfam was measured in human dermal fibroblasts. The H_2_O_2_ control showed lower mRNA expression of PGC-1*α* than that in normal cells. The expression of other transcription factors including ERR*α*, NRF1, and Tfam also decreased in the H_2_O_2_ control because these transcription factors are regulated via PGC-1*α* activation. However, KPE treatment elevated the mRNA expression of PGC-1*α* and its downstream genes, ERR*α*, NRF1, and Tfam. Based on these data, KPE stimulates the expression of mitochondrial biogenesis transcription factors by upregulating PGC-1*α* expression (Figure 7).

### 3.9. KPE Attenuates SA-*β*-Gal Activity In Vivo

**Figure 8:**
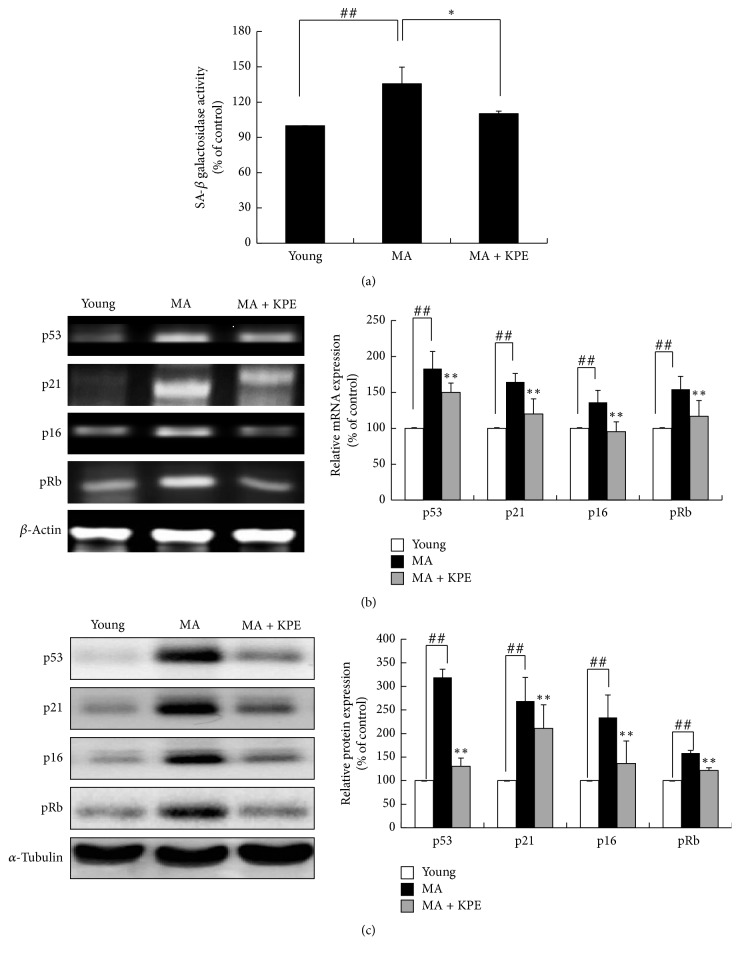
Effect of KPE on cellular senescence in vivo. (a) SA-*β*-gal activity was detected using a fluorescence reader at 365 nm excitation and 460 nm emission. (b) mRNA expression of p53, p21, p16, and pRb was evaluated via RT-PCR (c) The protein expression of p53, p21, p16, and pRb was evaluated via western blotting. Data are expressed as mean ± SD of five mice in each group. ^##^*p *< 0.01 compared to young mice; ^*∗*^*p* < 0.05 and ^*∗∗*^*p* < 0.01 compared to intrinsically MA mice.

In the intrinsically MA group, SA-*β*-gal activity is significantly elevated; however, this activity showed 25.5% reduction upon oral administration of KPE compared to that in the intrinsically MA group (Figure 8(a)). The results demonstrate that KPE treatment results in pronounced attenuation of age-related SA-*β*-gal activation, indicating its inhibitory effect on cellular senescence.

### 3.10. KPE Recovers Cell-Cycle Arrest In Vivo

Compared to young mice, intrinsically MA mice showed increased mRNA and protein levels of cell-cycle inhibitors, including p53, p21, p16, and pRb. In the KPE administered group, the p53, p21, p16, and pRb levels exhibited 33.1%, 44.4%, 40.8%, and 37.4% reduction, respectively, compared to those in the intrinsically MA group. The protein levels of cell-cycle inhibitors were also attenuated by KPE treatment (Figures 8(a) and 8(c)).

### 3.11. KPE Increases Mitochondrial Biogenesis In Vivo

**Figure 9:**
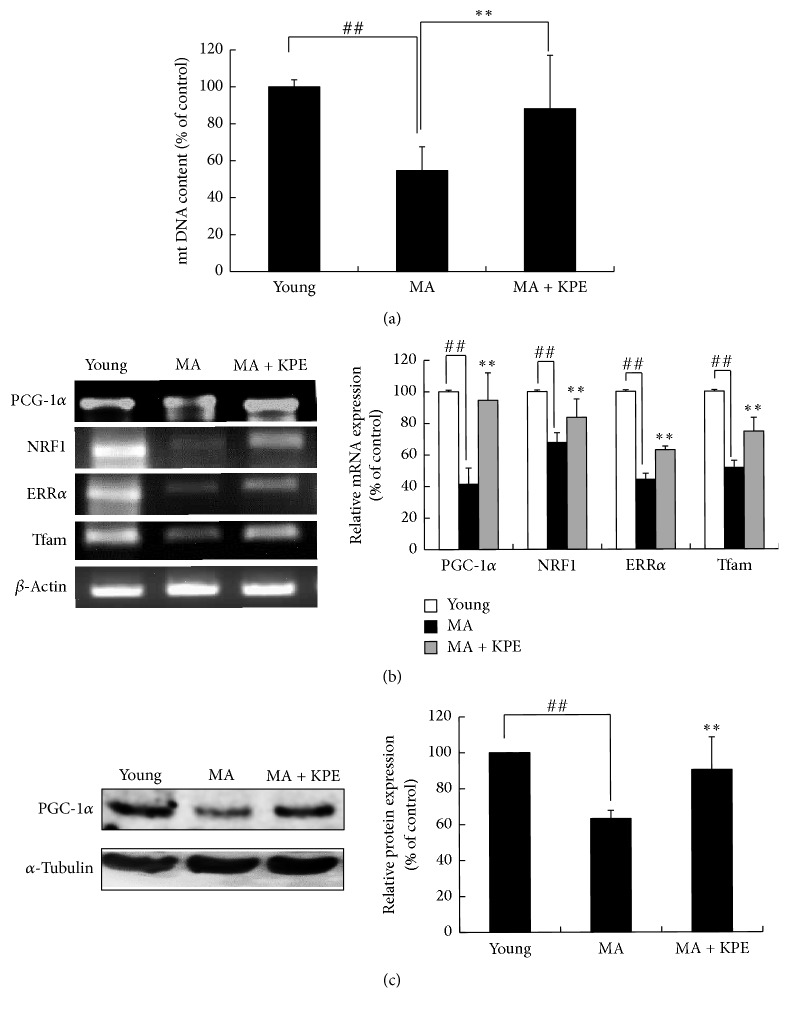
Effect of KPE on mitochondrial dysfunction in vivo. (a) Effect of KPE on mtDNA expression. (b) mRNA expression of PGC-1*α*, NRF-1, ERR*α*, and Tfam was evaluated via RT-PCR. (c) The protein expression of PGC-1*α* was evaluated via western blotting. Data are expressed as mean ± SD of five mice in each group. ^##^*p* < 0.01 compared to young mice; ^*∗∗*^*p* < 0.01 compared to intrinsically MA mice.

The level of PGC-1*α*, a crucial regulator of mitochondrial function as well as mitochondrial biogenesis, is decreased with aging [2]. The mRNA levels of PGC-1*α* and its downstream genes were reduced in intrinsically MA mice; however, KPE treatment upregulated the expression of these genes (Figures 9(b) and 9(c)), suggesting an enhancing effect of KPE on mitochondrial function in MA mice. The protein level of PGC-1*α* was consistent with its mRNA level. Consequently, KPE increased the mtDNA involved in mitochondrial function and biogenesis, supporting the observation that KPE improved mitochondrial function and biogenesis through PGC-1*α* stimulation (Figure 9(a)).

### 3.12. KPE Reduces Wrinkle Formation

**Figure 10:**
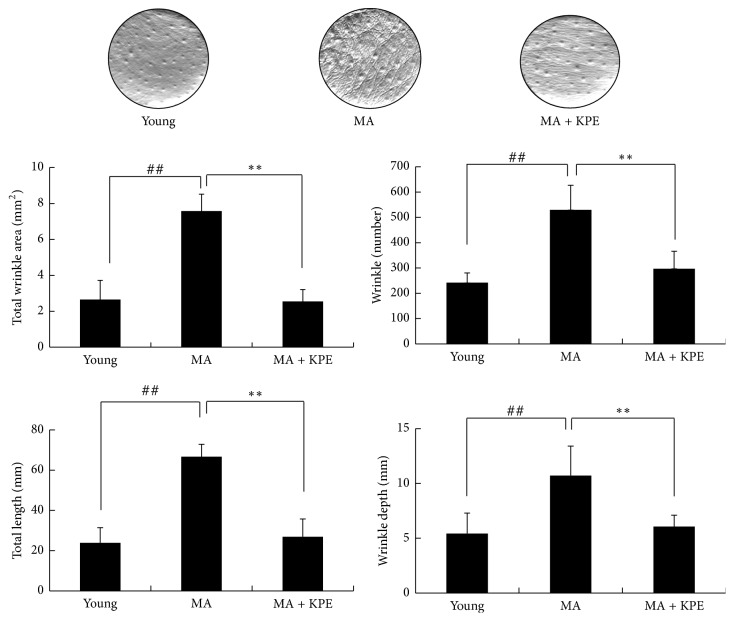
Effect of KPE on wrinkle values. Before the mice were killed, photographs and replica samples of the dorsal skin surface were taken. Wrinkle values were obtained from the skin replica analysis. Data are expressed as mean ± SD of five mice in each group. ^##^*p *< 0.01 compared to young mice; ^*∗∗*^*p* < 0.01 compared to intrinsically MA mice.

Wrinkle formation is a major characteristic of intrinsic skin aging [19]. Compared to young mice, intrinsically MA mice showed elevated wrinkle values, such as total area, number, length, and depth of wrinkles; however, the KPE administered group showed lower wrinkle values than those of the intrinsically MA group, suggesting that oral KPE administration alleviated wrinkle formation (Figure 10).

### 3.13. KPE Increases Skin Thickness

**Figure 11:**
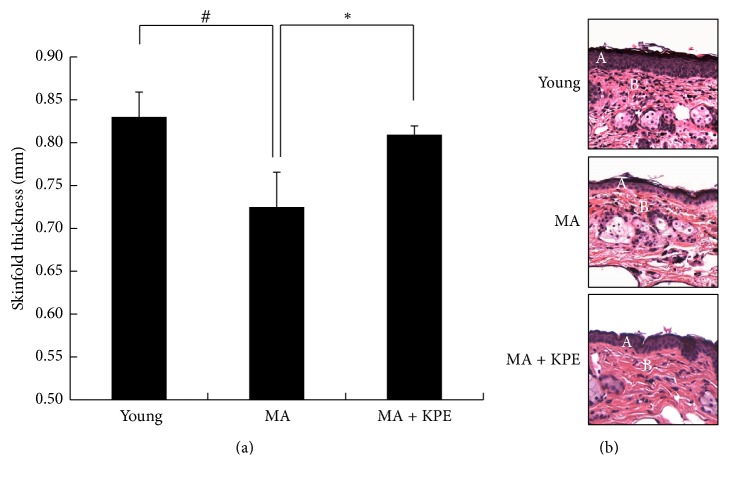
Effect of KPE on skinfold thickness. (a) Skinfold thickness was measured using a caliper mid-way between the neck and the hips at 24 weeks. (b) Skin tissue sections were stained with H&E. A, epidermis; B, dermis. Data are expressed as mean ± SD of five mice in each group. ^#^*p *< 0.05 compared to young mice; ^*∗*^*p* < 0.05 compared to intrinsically MA mice.

Photoaging is characterized by skin hypertrophy; in contrast, intrinsically aged skin shows skin thinning, also called skin atrophy [20]. Skin thickness and H&E staining were used to evaluate the extent of skin atrophy. The results showed the presence of skin atrophy in intrinsically MA mice; however, oral administration of KPE was found to attenuate skin atrophy (Figure 11).

### 3.14. KPE Inhibits Collagen Degradation

**Figure 12:**
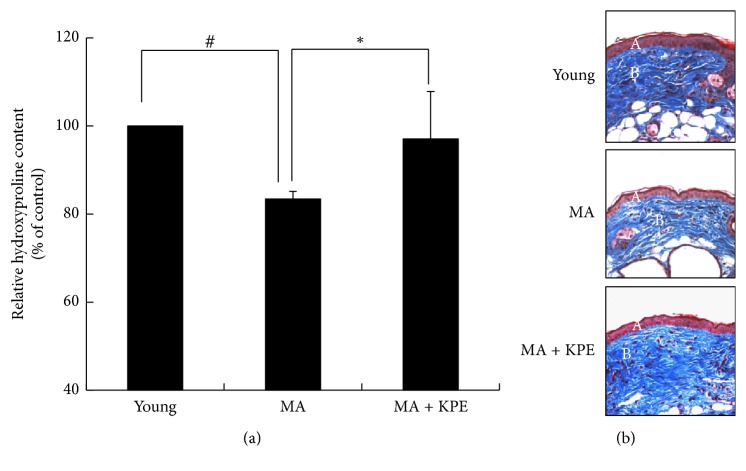
Effect of KPE on collagen content. (a) Hydroxyproline content was estimated after 24 weeks. (b) Skin tissue sections were stained with Masson's trichrome stain for collagen fibers. A, epidermis; B, dermis. Data are expressed as mean ± SD of five mice in each group. ^#^*p *< 0.05 compared to young mice; ^*∗*^*p* < 0.05 compared to intrinsically MA mice.

Collagen is the most abundant protein in the dermis. Aging induces loss of collagen, thus accelerating wrinkle formation [21]. The hydroxyproline content and M&T staining were used to measure the collagen content in skin. According to the results of the hydroxyproline assay and M&T staining, intrinsically MA mice showed low collagen content, whereas oral administration of KPE recovered the loss of collagen (Figure 12).

### 3.15. KPE Increases Elasticity

**Figure 13:**
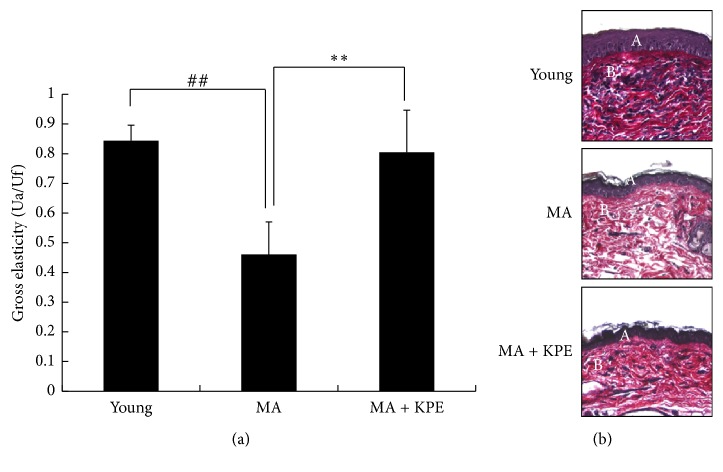
Effect of KPE on elasticity. (a) Effects of KPE on the gross elasticity (Ua/Uf) of skin were measured using Cutometer. (b) Skin tissue sections were stained with Verhoeff-van Gieson's stain. A, epidermis; B, dermis. Data are expressed as mean ± SD of five mice in each group. ^##^*p *< 0.01 compared to young mice; ^*∗∗*^*p* < 0.01 compared intrinsically to MA mice.

In intrinsically aged skin, elastic fiber atrophy occurs, thereby reducing skin elasticity [22]. Loss of collagen and elastic fibers results in wrinkling [20]. EVG staining showed that the elastic fiber content in intrinsically MA mice decreased; however, elastic fiber content of KPE administered mice was much higher as compared to that in the intrinsically MA mice. In addition, oral KPE administration increased the skin elasticity, indicating that skin elasticity improved after KPE treatment through reduction of age-related elastic fiber atrophy (Figure 13).

## 4. Discussion

Based on our results, KPE was found to attenuate cellular senescence in H_2_O_2_-treated fibroblasts and intrinsically MA mice by suppressing SA-*β*-gal activity and the expression of cell-cycle inhibitors. The restrained expression of cell-cycle inhibitors upon KPE treatment activates the E2F group, which is responsible for cell proliferation. In addition, KPE prevents cellular senescence by regulating the PI3K/AKT signaling pathway. Hyperactivation of AKT in the senescent state increases ROS generation. Recently, mTOR, one of the downstream regulators of AKT, has become a leading target for slowing aging and age-related diseases [23]. Well-known mTOR inhibitors such as rapamycin and resveratrol effectively suppress cellular senescence in vitro and in vivo [24]. Furthermore, AKT activation suppresses FoxO3a which decreases ROS levels and cellular senescence [7]. As shown in Figure 3, KPE regulates the H_2_O_2_-induced PI3K/AKT signaling pathway by affecting the levels of the downstream markers, FoxO3a and mTOR. These results indicate that KPE inhibits oxidative stress-induced cellular senescence by regulating the PI3K/AKT pathway in vitro; our study also demonstrates the inhibitory effect of KPE on cellular senescence using an in vivo model.

In pharmacological/nutritional approaches to longevity, there has been a great deal of interest in calorie restriction (CR) because it can increase the lifespan of model organisms [4]. Physiological changes under CR largely depend on SIRT1 expression, leading to resistance against senescence and aging. Accordingly, the search for natural CR mimetics has become a promising area in antiaging studies. Resveratrol is known as an effective CR mimetic. The senescence-reversing activity of resveratrol is mainly SIRT1-dependent [25, 26]. In the same vein, KPE considerably elevated expression of SIRT1 (Figure 4), indicating that KPE can also serve as an effective CR mimetic and is a potential natural antiaging agent.

Senescent cells secrete various factors known as the senescence-associated secretory phenotype (SASP), which affects neighboring cells. The SASP is a clear indication of senescence that is observed both in vitro and in vivo, including the presence of inflammatory cytokines, chemokines, proteases, growth factors, and insoluble compounds. In the present study, KPE effectively attenuated the senescence-associated inflammatory responses (Figure 5). Senescent fibroblasts stimulate neighboring cells by secreting inflammatory cytokines, including IL-6 and IL-8. In this state, normal neighboring cells can become dysfunctional, and premalignant cells can enter the malignant state, thus promoting cancer formation. Accordingly, senescence-associated chronic inflammation contributes to age-related diseases, a process known as inflamm-aging [2]. Our data suggest that KPE can serve as an effective antiaging agent through its anti-inflammatory activities.

In terms of mitochondrial dysfunction, H_2_O_2_-treated human skin fibroblasts and intrinsically MA hairless mice exhibited low levels of PGC-1*α* and its downstream genes such as ERR*α*, NRF-1, and Tfam. Reduced expression of PGC-1*α*, which is responsible for mitochondrial function and biogenesis, was recovered by KPE treatment (Figure 7). In addition, KPE reversed the decreased ATP levels and the increased ROS generation, which suggest mitochondrial dysfunction (Figure 6). A previous study has reported that age-related mitochondrial dysfunction alters the energy metabolism pathway. Senescent cells with mitochondrial dysfunction tend to produce energy via anaerobic glycolysis. In this process, advanced glycation end products (AGEs) are generated as by-products and negatively affect the extracellular matrix proteins, collagen, and elastin, leading to loss of contractile capacity [27]. Although cellular senescence and mitochondrial dysfunction are important targets of antiaging agents, natural products that have such efficacy in the skin are not as well known.

Several studies demonstrated that cellular senescence and mitochondrial dysfunction are closely interconnected via p53. Elevated p53 activation suppresses PGC-1*α*, resulting in mitochondrial dysfunction. On the contrary, mitochondrial dysfunction causes oxidative stress that induces cellular senescence. Both FoxO3, a downstream transcription factor of the PI3K/AKT signaling pathway, and SIRT1 have a relationship with p53 and PGC-1*α*. SIRT1 and FoxO3 increase the level of PGC-1*α*, whereas they decrease the p53 level [28, 29].

These two distinct causes of aging play an essential role in remodeling the ECM. During aging, the skin shows structural and functional changes [19]. The skin of intrinsically MA mice exhibited wrinkling, loss of elasticity, collagen and elastic fiber atrophy, and skin thinning. These changes were diminished by the oral administration of KPE (Figures 10–13). The aging process drives the degeneration of collagen and elastic fibers, which is generally considered to be involved in wrinkle formation, loss of elasticity, and skin thinning. Accordingly, the regulation of collagen and elastic fiber degeneration is essential to prevent the intrinsic skin aging phenotype. Histological staining indicated increased collagen and elastic fiber content in KPE administered mice.

Our previous study on the antiphotoaging effect of KPE showed decreased wrinkling and loss of collagen fiber in KPE (100 or 200 mg/kg/day)-treated UVB-irradiated hairless mice [30]. Because the high-dose KPE group gave better preventive effect on UVB-induced photoaging than that of lower-dose KPE group, we use the dose of KPE 200 mg/kg/day in this research. KPE prevents both extrinsic and intrinsic aging process in hairless mice. However, when hairless mice were subjected to UVB irradiation, their skin thickness was significantly increased [30] in association with an inflammatory response. UVB-induced skin inflammation elicits epidermal proliferation leading to skin thickening [30]. In contrast to that in extrinsic aging, the skin thickness of intrinsically aged mice decreased. Skin thinning in intrinsic skin aging is reportedly associated with the degeneration of collagen and elastic fibers. In addition, several studies have supported an intimate relationship between cellular senescence and skin atrophy. An investigation using superoxide dismutase 2- (Sod2-) deficient mice has verified that mitochondrial oxidative stress promotes cellular senescence and contributes to the skin thinning aging phenotype [20, 31].

## 5. Conclusion

In conclusion, KPE delays intrinsic skin aging process by inhibiting cellular senescence and mitochondrial dysfunction. KPE does not only attenuate cellular senescence through inhibition of the p53/p21, p16/pRb, and PI3K/AKT signaling pathways but also improve mitochondrial biogenesis through PGC-1*α* stimulation in H_2_O_2_-exposed human dermal fibroblasts and MA hairless mice. Consequently, KPE prevents wrinkle formation, skin atrophy, and loss of elasticity by increasing collagen and elastic fibers in MG hairless mice. Therefore, KPE can serve as an antiaging nutraceutical and cosmeceutical agent.
